# Beneficial Effects of Gegen Qinlian Decoction and Its Food–Medicine Homologous Alternative Formulas Against Type 2 Diabetes Mellitus: Insights from Multi-Omics Analysis

**DOI:** 10.3390/ph19040530

**Published:** 2026-03-25

**Authors:** Yao Chen, Dandan Ma, Qiuming Chen, Maomao Zeng, Jie Chen, Zhiyong He

**Affiliations:** 1 State Key Laboratory of Food Science and Resources, Jiangnan University, Wuxi 214122, China; 2 School of Food Science and Technology, Jiangnan University, Wuxi 214122, China

**Keywords:** Gegen Qinlian Decoction, food-medicine homologous formula, type 2 diabetes mellitus, multi-omics, antidiabetic effect

## Abstract

**Background/Objectives:** Herbal formulas are unsuitable for routine dietary use. This study evaluates Qige Pipa Decoction (QPD), a food–medicine homologous formula containing edible components, comparing its anti-diabetic effects with the classic Gegen Qinlian Decoction (GQD) to explore its potential as a sustainable dietary intervention for T2DM. **Methods:** T2DM mice received QPD, GQD, or metformin for 6 weeks. Parameters included glycemic control, histopathology, gut microbiota (16S rRNA), serum metabolomics, liver transcriptomics, and chemical profiling (UPLC-Q-TOF-MS). **Results:** Both formulas comparably improved glycemia and insulin resistance. QPD uniquely enriched beneficial gut bacteria (e.g., *Roseburia*) and suppressed pro-inflammatory taxa. Metabolomics revealed decreased Carnitine C20:1 and increased phospholipids in the QPD group. Transcriptomics showed QPD enriched the AGE-RAGE signaling pathway. Chemically, QPD showed relatively higher signal intensities for glycosides and organic acids, while GQD showed relatively higher signal intensities for alkaloids. **Conclusions:** QPD exhibits anti-diabetic efficacy similar to GQD but through distinct regulatory mechanisms. Its food-medicine homologous composition provides a theoretical rationale for its exploration as a sustained dietary adjunct. However, the absence of safety biomarkers in this study precludes definitive conclusions regarding long-term tolerability, necessitating dedicated toxicological assessment in future trials.

## 1. Introduction

Type 2 diabetes mellitus (T2DM) has emerged as a global pandemic, posing a severe threat to public health due to its high prevalence and associated complications [1]. Its pathogenesis is complex, involving insulin resistance, β-cell dysfunction, and chronic low-grade inflammation [2,3]. In recent years, the intricate interplay between gut microbiota dysbiosis, host metabolism disruption, and multi-organ communication (especially the gut-liver axis) has been recognized as a pivotal contributor to the development and progression of T2DM [4,5]. This systemic perspective underscores the limitations of single-target pharmacological interventions and highlights the potential of holistic therapeutic approaches, such as traditional Chinese medicine (TCM), which aim to restore balance through multi-component, multi-target mechanisms [6,7].

Gegen Qinlian Decoction (GQD), a classic TCM formula recorded in Treatise on Cold Damage Diseases, consists of four key herbs: *Pueraria lobata* (Gegen, 8 parts), *Coptidis Rhizoma* (Huanglian, 3 parts), *Scutellaria baicalensis* (Huangqin, 3 parts), and *Glycyrrhiza uralensis* (Gancao, 2 parts). It has been clinically used and empirically proven effective in alleviating diabetic symptoms, particularly those associated with damp-heat syndrome [8]. Modern pharmacological studies have confirmed that GQD can lower blood glucose, improve insulin sensitivity, and modulate gut microbiota [9,10]. However, the routine use of some herbal constituents in GQD, such as *Scutellaria baicalensis* (Huangqin) and *Coptidis Rhizoma* (Huanglian), which possess a “cold” property, may cause gastrointestinal discomfort in long-term management [11,12]. This concern aligns with the TCM principle of “treating disease by considering the constitution,” suggesting a need for safer, more tolerable formulations for chronic conditions like T2DM. Inspired by the concept of “food and medicine homologous” (FMH) substances, substituting traditional herbs with FMH alternatives offers a promising strategy to enhance safety and patient compliance while preserving efficacy. In the classic GQD formula, *Scutellaria baicalensis* (Huangqin) and Coptidis *Rhizoma* (Huanglian) are pivotal herbs that clear “damp-heat” and “fire”, respectively, which are core pathological factors in T2DM from the TCM perspective. However, their pronounced “cold” property can cause gastrointestinal discomfort with long-term use. To address this, we sought FMH alternatives that possess comparable efficacy in managing the core symptoms (damp-heat, hyperglycemia) but with a milder property profile suitable for chronic dietary intervention. *Astragalus membranaceus* (Huangqi) was selected to substitute for *Scutellaria baicalensis*. While both herbs target T2DM, their mechanisms differ. Modern research indicates *Scutellaria baicalensis* primarily exerts anti-inflammatory and antioxidant effects via its flavonoids [13], whereas *Astragalus membranaceus* acts as a qi-tonifying herb, with its polysaccharides and saponins demonstrating potent effects in improving insulin sensitivity, protecting pancreatic β-cells, and modulating immunity [14]. Likewise, *Coptidis Rhizoma*, a representative alkaloid-rich medicinal herb in GQD, has been widely reported to exert anti-diabetic effects through multiple pathways, including regulation of glucose metabolism, inflammation, and gut microbiota [15]. In contrast, *Eriobotrya japonica Thunb.* leaves, a common dietary and medicinal material, contain triterpenoids and flavonoids that have demonstrated significant anti-diabetic properties, including inhibition of carbohydrate-digesting enzymes (α-glucosidase, α-amylase), improvement of glucose uptake, and anti-inflammatory effects [16]. Based on this evidence, we designed a novel FMH-alternative formula, termed Qige Pipa Decoction (QPD), in which *Scutellaria baicalensis* and *Coptidis Rhizoma* in the original GQD were replaced by *Astragalus membranaceus* and *Eriobotrya japonica Thunb.* leaves, respectively, on a gram-for-gram basis to maintain the original formula’s proportional framework for herbal formulation consistency. Notably, this gram-for-gram substitution was applied for formulation standardization only and does not imply phytochemical, functional, or pharmacological equivalence between the original and substitute herbs. The systemic therapeutic effects and underlying mechanisms of this newly designed FMH formula, and whether it retains GQD’s anti-diabetic efficacy via distinct pathways, remain largely unknown.

Therefore, this study aimed to comprehensively evaluate and compare the anti-diabetic effects of GQD and its FMH-alternative formula QPD in a T2DM animal model. We employed a multi-omics integration strategy, combining 16S rRNA gene sequencing for gut microbiota analysis, untargeted serum metabolomics, and hepatic transcriptomics. We hypothesized that QPD would exert comparable glycemic control and multi-system regulatory effects to GQD, potentially through distinct yet converging pathways involving the remodeling of gut microbial communities, correction of metabolic disturbances, and regulation of key gene networks in the liver. This work not only provides scientific evidence for the innovation of TCM formulas based on the FMH concept but also offers novel insights into the systemic mechanisms of herbal medicine against T2DM.

## 2. Results

### 2.1. Effects of QPD and GQD on Glucose Metabolism and Histopathology in T2DM Mice

Glucose metabolism disorders and insulin resistance are central to the pathogenesis and progression of T2DM, often accompanied by target organ damage including hepatic steatosis and pancreatic tissue injury [17]. In the present study, a T2DM mouse model was established to systematically evaluate the therapeutic effects of QPD on glucose metabolism and histopathological changes in the liver and pancreas, with parallel comparison to the classic formula GQD.

**Figure 1:**
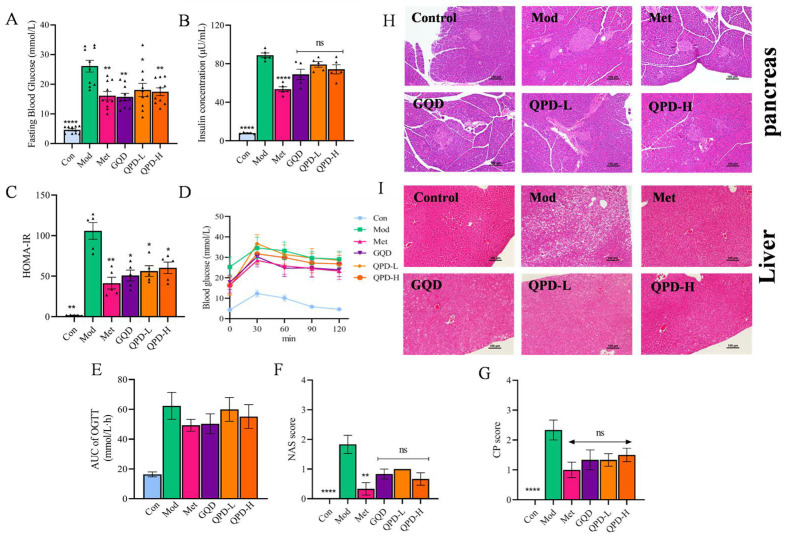
QPD and GQD alleviate glucose metabolic disorders and histopathological injuries in T2DM mice. (**A**) Fasting blood glucose levels in each group. (**B**) Fasting Serum insulin concentrations. (**C**) Homeostatic model assessment of insulin resistance (HOMA-IR). (**D**) Oral glucose tolerance test (OGTT) curves. Data were analyzed by two-way repeated-measures ANOVA with Bonferroni’s post hoc test. (**E**) Area under the curve (AUC) of OGTT. (**F**) NAFLD activity score (NAS). (**G**) CP score. (**H**) Pancreatic tissue section (**I**) Liver tissue section. Data are presented as mean ± SEM. Statistical analysis was performed using one-way ANOVA followed by Dunnett’s multiple comparisons test for panels (**A**–**C**,**E**–**G**), with the Model group as the control. * *p* < 0.05, ** *p* < 0.01, **** *p* < 0.0001 vs. Mod; ns indicates not significant. Each triangle = one mouse.

FBG levels were significantly elevated in the model (Mod) group compared to the control (Con) group (*p* < 0.0001, Figure 1A). Following drug intervention, FBG levels were markedly reduced in the Met, GQD, QPD-L, and QPD-H groups relative to the Mod group (*p* < 0.05 or *p* < 0.01), with the glucose-lowering effect of QPD-H being comparable to those of GQD and Met. The Mod group exhibited significantly higher serum insulin concentrations than the Con group (*p* < 0.0001, Figure 1B); however, neither Met, GQD, nor QPD at any dose significantly reduced insulin levels. Insulin resistance, assessed by the homeostatic model assessment for insulin resistance (HOMA-IR), was markedly elevated in the Mod group (*p* < 0.0001, Figure 1C), whereas all treatment (Met, GQD, QPD-L, and QPD-H) groups significantly lowered HOMA-IR (*p* < 0.05 or *p* < 0.01), suggesting improved insulin sensitivity. The oral glucose tolerance test (OGTT) revealed that blood glucose levels at all time points and the area under the curve (AUC) were significantly higher in the Mod group than in the Con group (Figure 1D,E). Treatment with Met, GQD, and both doses of QPD effectively reduced OGTT glucose levels at each time point and de-creased the corresponding AUC.

In the Mod group, the NAS score was significantly elevated compared to the Con group [18] (*p* < 0.0001, Figure 1F). H&E staining revealed diffuse hepatocellular steatosis and disrupted hepatic architecture in model mice (Figure 1I). Following intervention, all treatment groups exhibited a trend toward reduced NAS scores (Figure 1F), although these differences did not reach statistical significance. Histological examination showed modest improvements in steatosis and structural organization across treatment groups. Regarding pancreatic pathology, model mice displayed marked islet atrophy, reduced islet cell numbers, and disorganized cellular arrangement [19] (Figure 1G). After treatment, all intervention groups showed a trend toward improved islet morphology and cellular organization, though no statistically significant differences in CP score were observed (Figure 1H).

### 2.2. Effects of QPD and GQD on Gut Microbiota in T2DM Mice

**Figure 2:**
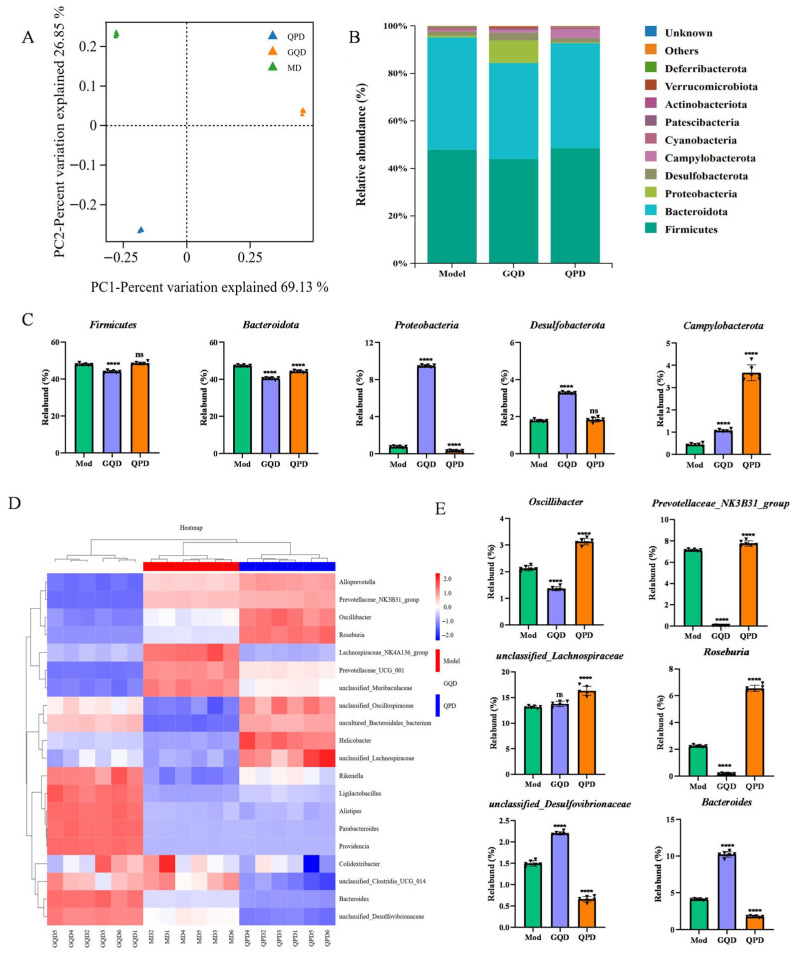
QPD and GQD differentially modulate gut microbiota composition in T2DM mice. (**A**) Principal coordinates analysis (PCoA) of gut microbiota structure among Mod, GQD, and QPD groups. (**B**) Relative abundance of gut microbiota at the phylum level. (**C**) Comparative analysis of key phyla abundances across groups. (**D**) Heatmap showing the abundance of differentially abundant microbial genera among groups. (**E**) Relative abundances of representative beneficial bacterial genera, including *Oscillibacter*, *Prevotellaceae_NK3B31_group*, *unclassi-fied_Lachnospiraceae*, and *Roseburia*. Data are presented as mean ± SEM. Statistical significance in Panels (**C**,**E**) was assessed by Kruskal–Wallis test with Dunn’s post hoc test and FDR correction for multiple comparisons against the Model group. Beta-diversity clustering in Panel (**A**) was evaluated by PERMANOVA (R^2^ = 0.42, *p* = 0.001) and ANOSIM (R = 0.68, *p* = 0.001). **** *p* < 0.0001 vs. Mod; ns indicates not significant. Each triangle = one mouse.

Gut microbiota dysbiosis is a critical driver in the pathogenesis and progression of T2DM [20]. Principal coordinates analysis (PCoA) based on Bray–Curtis dissimilarity revealed distinct differences in gut microbial community composition among the Mod, GQD, and QPD groups (Figure 2A). The result of PCoA revealed distinct clustering of gut microbial communities among the Mod, GQD, and QPD groups (Figure 2A). At the phylum level (Figure 2B), *Firmicutes* and *Bacteroidota* were the dominant phyla across all groups. Compared to the Mod group, the GQD group exhibited significantly reduced abundances of both *Firmicutes* and *Bacteroidota* (*p* < 0.0001). In the QPD group, no significant difference was observed in *Firmicutes* abundance, whereas *Bacteroidota* abundance was markedly decreased (*p* < 0.0001). Additionally, GQD treatment led to significant increases in the abundances of *Proteobacteria*, *Desulfobacterota*, and *Campylobacterota* (*p* < 0.0001), while QPD treatment specifically elevated *Campylobacterota* abundance (*p* < 0.0001) (Figure 2C).

Heatmap analysis demonstrated distinct alterations in the abundance of significantly differentiated bacterial taxa among groups, with the GQD and QPD groups ex-hibiting similar clustering patterns but clearly separated from the Mod group (Figure 2D). At the genus level, compared to the model group, the QPD group showed significantly increased abundances of *Oscillibacter*, *Prevotellaceae_NK3B31_group*, *unclassified_Lachnospiraceae*, and *Roseburia* (*p* < 0.0001), whereas the GQD group exhibited marked reductions in these bacteria (*p* < 0.0001, Figure 2E). Conversely, the abundances of *unclassified_Desulfovibrionaceae* and *Bacteroides* were significantly decreased in the QPD group (*p* < 0.0001) but markedly increased in the GQD group (*p* < 0.0001).

### 2.3. Effects of QPD and GQD on Serum Metabolome in T2DM Mice

**Figure 3:**
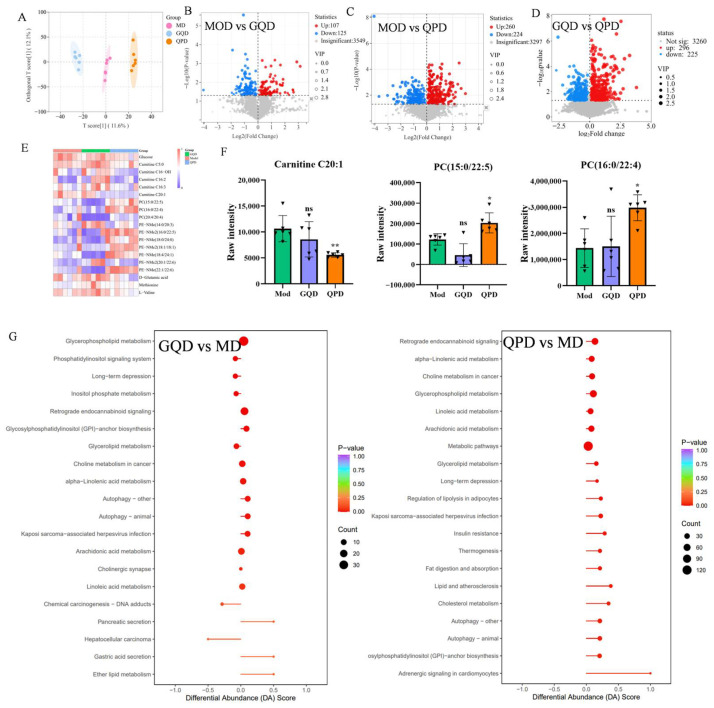
QPD and GQD differentially modulate serum metabolomic profiles in T2DM mice. (**A**) Orthogonal partial least squares discriminant analysis (OPLS-DA) score plot showing distinct metabolic clustering among Mod, GQD, and QPD groups. (**B**–**D**) Volcano plots of differential metabolites in GQD vs. Mod, QPD vs. Mod and GQD vs. QPD. (**E**) Heatmap showing the relative abundances of key differential metabolites across groups. (**F**) Quantitative analysis of key differential metabolites in each group. (**G**) KEGG pathway enrichment analysis of differential metabolites in GQD vs. Mod, QPD vs. Mod and GQD vs. QPD. Data are presented as mean ± SEM. Panel (**F**): Statistical analysis was performed using one-way ANOVA followed by Tukey’s multiple comparisons test to evaluate differences among all groups. * *p* < 0.05, ** *p* < 0.01 vs. Mod; ns indicates not significant. Each triangle=one mouse. Panels (**B**–**D**) (Volcano plots): Differential metabolites were identified using Student’s *t*-test with FDR correction (Benjamini–Hochberg method) for multiple comparisons between groups. OPLS-DA (Panel (**A**)) was conducted using ropls package.

Serum metabolomic disturbance is a key characteristic of T2DM [21]. OPLS-DA analysis (Figure 3A) revealed distinct separation of serum metabolic profiles among the Mod, GQD, and QPD groups. The OPLS-DA model showed strong explanatory and predictive ability with R^2^Y = 0.996 and Q^2^ = 0.688. A permutation test further confirmed that the model was robust and not overfitted (Figure S2). Based on the criteria of VIP > 1.0, |log2FC| > 0.263, and FDR-corrected *p* < 0.05, Volcano plot analysis (Figure 3B–D) showed that, compared with the model group, a total of 232 differentially expressed metabolites (107 upregulated, 125 downregulated) were identified in the GQD group, while 484 differentially expressed metabolites (260 upregulated, 224 downregulated) were identified in the QPD group. Furthermore, 521 differentially expressed metabolites were identified between the GQD and QPD groups. According to MSI guidelines, these metabolites were annotated as Level 2.

The heatmap in Figure 3E visually illustrates the clustering patterns of differential metabolites associated with glucose and lipid metabolism across groups. Further statistical analysis of key differential metabolites (Figure 3F) revealed that, compared to the mod group, the QPD group exhibited significantly reduced abundance of Carnitine C20:1, along with markedly increased levels of phospholipid metabolites including PC (15:0/22:5) and PC (16:0/22:4) (*p* < 0.05 or *p* < 0.01). The regulatory trends of these metabolites in the GQD group differed from those observed in the QPD group. Pathway enrichment analysis of differential metabolites (Figure 3G) revealed distinct metabolic pathways modulated by the two formulas. In the GQD group, differential metabolites were primarily enriched in glycerophospholipid metabolism, the phosphatidylinositol signaling system, and long-term depression. In contrast, differential metabolites in the QPD group were mainly enriched in retrograde endocannabinoid signaling, alpha-linolenic acid metabolism, choline metabolism in cancer, insulin resistance, and thermogenesis.

### 2.4. Effects of QPD and GQD on Liver Transcriptome in T2DM Mice

**Figure 4:**
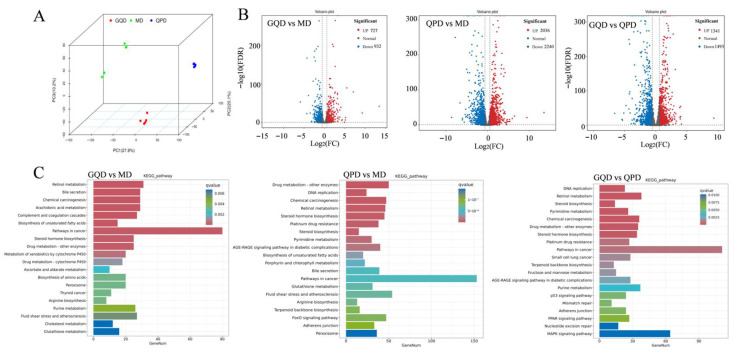
QPD and GQD differentially regulate hepatic transcriptomic profiles in T2DM mice. (**A**) PCA score plot among Mod, GQD, and QPD groups based on variance-stabilized transformation (VST) normalized counts. (**B**) Volcano plots of differentially expressed genes in GQD vs. Mod, QPD vs. Mod, and GQD vs. QPD. Differential expression analysis was performed using DESeq2 with Wald test and Benjamini–Hochberg adjustment; significance thresholds were set at |log2 fold change| > 1 and adjusted FDR ≤ 0.05. (**C**) KEGG pathway enrichment analysis of differentially expressed genes in GQD vs. Mod, QPD vs. Mod, and GQD vs. QPD. KEGG pathway enrichment analysis was conducted using hypergeometric test with FDR correction (*p* < 0.05 considered significant).

The liver undergoes transcriptomic reprogramming that critically drives the pro-gression of diabetes [22]. Clear separation of hepatic transcriptional profiles among the model, GQD, and QPD groups was observed in principal component analysis (Figure 4A), with samples from each group forming distinct clusters. Volcano plot analysis (Figure 4B) revealed that, relative to the model group, GQD intervention yielded 1659 differen-tially expressed genes (727 upregulated, 932 downregulated). In comparison, QPD induced 4276 differentially expressed genes (2036 upregulated, 2240 downregulated). Notably, 2834 genes (1341 upregulated, 1493 downregulated) were differentially expressed between the GQD and QPD groups.

KEGG pathway enrichment analysis (Figure 4C) demonstrated that DEGs modu-lated by GQD were primarily enriched in retinol metabolism, bile secretion, chemical carcinogenesis, arachidonic acid metabolism, and cancer-related pathways. By contrast, DEGs regulated by QPD were predominantly associated with drug metabolism, DNA replication, chemical carcinogenesis, steroid hormone biosynthesis, and the AGE-RAGE signaling pathway in diabetic complications. Furthermore, genes differentially expressed between the two treatment groups were mainly enriched in DNA replication, retinol metabolism, pyrimidine metabolism, and cancer pathways.

**Figure 5:**
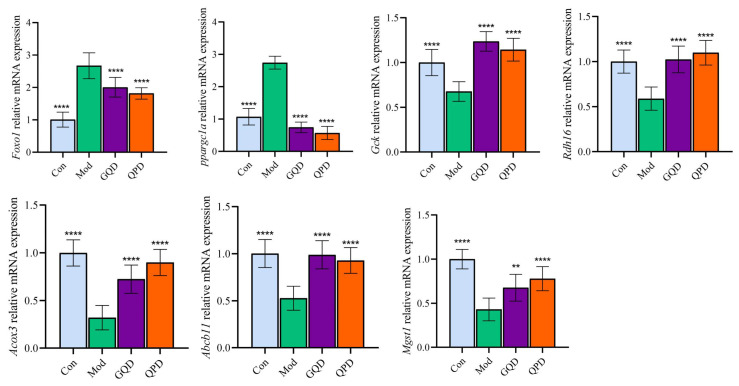
RT-qPCR validation of representative hepatic genes regulated by GQD and QPD in T2DM mice. Data are presented as mean ± SEM. Statistical significance was assessed using one-way ANOVA followed by an appropriate post hoc test. ** *p* < 0.01, **** *p* < 0.0001 vs. Mod.

To validate the reliability of the transcriptomic results, seven representative DEGs, namely *Foxo1*, *Ppargc1a*, *Gck*, *Rdh16*, *Acox3*, *Abcb11*, and *Mgst1*, were selected for qPCR analysis (Figure 5). These genes were associated with several enriched pathways identified by KEGG analysis. Specifically, Rdh16 and Abcb11 were related to retinol metabolism and bile secretion, respectively, Mgst1 was involved in glutathione metabolism and chemical carcino-genesis-related processes, Foxo1 was associated with both diabetes-related glucose metabolic signaling and the AGE-RAGE signaling pathway, Ppargc1a and Gck were linked to glucose metabolism-related pathways, and Acox3 was associated with fatty acid metabolism and peroxisome-related pathways. The qPCR results were generally consistent with the RNA-seq data. Compared with the control group, the model group exhibited significantly increased expression of Foxo1 and Ppargc1a, whereas *Gck*, *Rdh16*, *Acox3*, *Abcb11*, and *Mgst1* were markedly downregulated. Both GQD and QPD treatment significantly reversed these abnormal expression patterns, further confirming the reliability of the transcriptomic analysis.

### 2.5. Integrated Multi-Omics Correlation Analysis Linking Gut Microbiota, Serum Metabolites, and Hepatic Gene Expression

**Figure 6:**
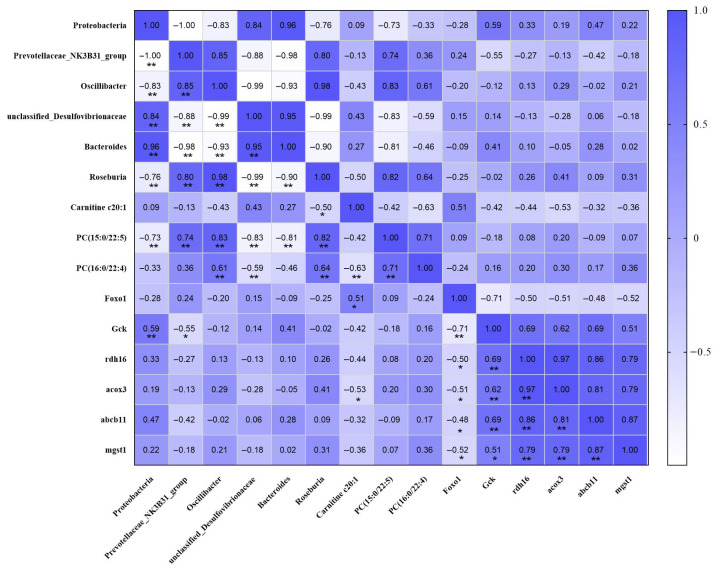
Integrated correlation heatmap of gut microbiota, serum metabolites, and hepatic gene expression. Pearson correlation analysis was performed across all samples. Red and blue colors indicate positive and negative correlations, respectively. * *p* < 0.05, ** *p* < 0.01.

To further integrate the multi-omics data, Pearson correlation analysis was performed among differential gut microbial taxa, representative serum metabolites, and hepatic genes across all samples (Figure 6). The integrated correlation heatmap revealed coordinated associations among gut microbiota, serum metabolites, and host hepatic gene expression. At the microbiota–metabolite level, Roseburia was significantly negatively correlated with Carnitine C20:1, but positively correlated with PC (15:0/22:5) and PC (16:0/22:4). Similarly, Prevotellaceae_NK3B31_group and Oscillibacter showed significant positive correlations with PC (15:0/22:5), whereas Proteobacteria, Bacteroides, and unclassified_Desulfovibrionaceae were negatively correlated with this metabolite. In addition, Carnitine C20:1 was significantly negatively correlated with PC (16:0/22:4). At the metabolite–gene level, Carnitine C20:1 was positively correlated with Foxo1 and negatively correlated with acox3. Meanwhile, Foxo1 showed significant negative correlations with Gck, rdh16, acox3, abcb11, and mgst1, whereas Gck was positively correlated with rdh16, acox3, abcb11, and mgst1. These results suggest coordinated associations linking gut microbial alterations, serum metabolic changes, and hepatic transcriptional responses.

### 2.6. Chemical Composition Analysis of QPD and GQD

**Figure 7:**
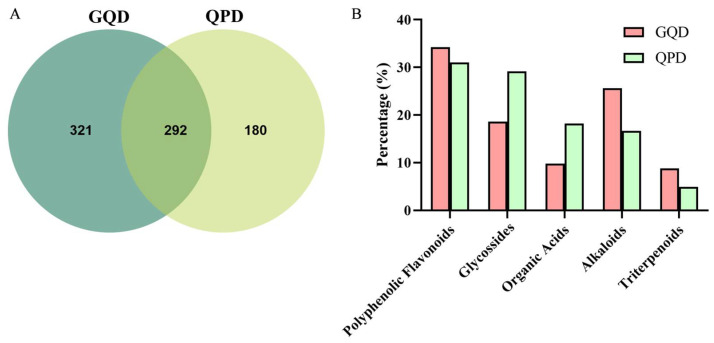
Semi-quantitative chemical composition analysis of QPD and GQD based on UPLC-Q-TOF-MS relative signal intensities. (**A**) Venn diagram illustrating the number of shared and unique compounds identified in QPD and GQD. (**B**) Classification and relative signal abundance proportions of major chemical constituents in QPD and GQD.

Venn diagram analysis (Figure 7A) revealed a total of 793 compounds identified across both formulas, comprising 292 shared constituents, 321 GQD-specific components, and 180 QPD-specific components. Classification and statistical analysis of the identified compounds (Figure 7B) demonstrated that polyphenolic flavonoids constituted the predominant chemical class in both formulas, accounting for approximately 34% in GQD and 31% in QPD. GQD exhibited relatively higher signal intensities for alkaloids and triterpenoids compared to QPD, whereas QPD showed relatively higher signal intensities of glycosides, as well as organic acids. Semi-quantitative comparison of ion abundances (Table 1) further elucidated these compositional distinctions. GQD was characterized by strong signal intensities for alkaloids including berberine, palmatine, and moupinamide, along with flavonoid glycosides such as baicalin and wogonoside. In contrast, QPD was predominantly enriched in isoflavonoids including daidzin, mirificin, and daidzein, as well as triterpenoids such as glycyrrhizic acid and ganoderic acid S.

## 3. Discussion

This study systematically evaluated the therapeutic effects of QPD in a mouse model of T2DM and conducted a parallel comparison with the classic formula GQD. The results demonstrated that the overall efficacy of QPD in ameliorating disorders of glucose and lipid metabolism, insulin resistance, and histopathological damage in the liver and pancreas was comparable to that of GQD. However, further integrated analyses of the gut microbiota, serum metabolome, and hepatic transcriptome revealed significant differences in their underlying mechanisms of action, which are closely associated with their distinct chemical compositions.

The present study showed that QPD significantly reduced fasting blood glucose, improved oral glucose tolerance, and lowered HOMA-IR in T2DM mice. The reduction in HOMA-IR observed in the QPD groups was comparable to that achieved by GQD, which is consistent with previous reports that GQD exerts hypoglycemic effects partly through improving insulin sensitivity [23]. These findings suggest that QPD may ameliorate glucose metabolism disorders through a similar regulatory pattern. Notably, fasting insulin levels were not significantly altered by either GQD or QPD, despite the marked reduction in HOMA-IR. This finding suggests that the beneficial effects of GQD and QPD on glucose metabolism may not primarily depend on changes in insulin secretion, but rather on improvements in insulin sensitivity and systemic metabolic regulation. Histopathological examination revealed directional improvements in hepatic steatosis (reduced NAS scores) and islet architecture (improved CP scores) following QPD intervention; however, these changes failed to achieve statistical significance (*p* > 0.05). These observational trends suggest that QPD may potentially exert mild beneficial effects on diabetes-associated hepatic injury, possibly through the modulation of lipid metabolic processes, and may have a tentative protective potential for pancreatic islet structural integrity, though these putative effects require further validation with larger sample sizes and longer intervention durations to establish statistical robustness.

Analysis of the gut microbiota uncovered a fundamental divergence in the regulatory patterns of QPD and GQD. At the phylum level, QPD and GQD already exhibited distinct microbial remodeling patterns. QPD markedly reduced the relative abundance of Proteobacteria, whereas GQD significantly increased this phylum. Numerous studies have endorsed that an expansion (or bloom) of Proteobacteria in the gut reflects dysbiosis or an unstable gut microbial community structure, and this expansion is frequently associated with metabolic disorders and chronic intestinal inflammation [24]. Therefore, the reduction in Proteobacteria in the QPD group may reflect an improvement in intestinal microecological balance, whereas its enrichment in the GQD group suggests that the microbial remodeling induced by GQD differs substantially from that of QPD. PCoA and heatmap analyses indicated that both formulas significantly remodeled the gut microbial structure in diabetic mice, but exhibited opposite regulatory trends at the genus level. QPD significantly en-riched known short-chain fatty acid (SCFA)-producing bacteria such as *Oscillibacter*, *Prevotellaceae_NK3B31_group*, and *Roseburia*. Specifically, *Prevotellaceae_NK3B31_group* degrades complex carbohydrates to produce propionate, which regulates gluconeogenesis and insulin sensitivity [25]. *Roseburiais,* a primary producer of butyrate, which serves as an energy source for colonocytes, enhances intestinal barrier function, and reduces endotoxemia, thereby mitigating the systemic chronic inflammation that is a key driver of insulin resistance [26]. Consequently, the enrichment of these beneficial bacteria by QPD may improve host metabolism by increasing intestinal SCFA levels. Conversely, QPD significantly suppressed the abundance of potentially pro-inflammatory or pathogenic bacteria such as *unclassified_Desulfovibrionaceae* and *Bacteroides*. Members of the *Desulfovibrionaceae* family produce hydrogen sulfide, which damages the intestinal epithelial barrier and leads to endotoxemia and chronic low-grade inflammation [27]. Certain *Bacteroidesspecies* can generate pro-inflammatory lipopolysaccharides under specific conditions, exacerbating insulin resistance [28]. Notably, GQD exhibited an almost completely opposite regulatory direction for these microbial taxa. This stark contrast in reshaping the gut microecology strongly suggests that QPD and GQD exert their anti-diabetic effects by targeting distinct microbial communities. QPD primarily improves intestinal microecological balance and alleviates chronic low-grade inflammation by enriching SCFA producers and suppressing pro-inflammatory bacteria, whereas GQD appears to operate through a different regulatory network. It should be noted that these taxon-level microbial differences were identified using conventional statistical tests with FDR correction, which may not fully account for the compositional nature of 16S rRNA sequencing data. Therefore, the genus- and species-level changes described above should be interpreted with appropriate caution, although the overall trend is supported by the community-level analyses and the integrated multi-omics results.

Serum untargeted metabolomics findings further support the mechanistic differences between the two formulas. Compared to the model group, QPD intervention significantly reduced serum levels of the long-chain acylcarnitine, Carnitine C20:1. Long-chain acylcarnitines typically accumulate under insulin-resistant conditions, and their accumulation can induce lipotoxicity and worsen insulin signaling impairment [29]. The specific reduction in this metabolite suggests that QPD may enhance mitochondrial fatty acid β-oxidation, reducing the aberrant accumulation of lipid intermediates and thereby improving insulin sensitivity. Simultaneously, QPD markedly increased the levels of polyunsaturated phosphatidylcholines such as PC (15:0/22:5) and PC (16:0/22:4). These phospholipids, enriched with polyunsaturated fatty acids, help increase cell membrane fluidity and promote insulin receptor signaling efficiency [30]. Pathway enrichment analysis revealed that differential metabolites regulated by QPD were primarily involved in retrograde endocannabinoid signaling, alpha-linolenic acid metabolism, choline metabolism in cancer, insulin resistance, and thermogenesis—pathways closely associated with diabetic pathophysiology. In contrast, metabo-lites regulated by GQD were mainly enriched in glycerophospholipid metabolism and the phosphatidylinositol signaling system. This indicates that QPD ameliorates diabetes-related metabolic disorders principally through the regulation of lipid metabolism, energy metabolism, and inflammation-associated pathways, a pattern distinct from that of GQD.

Hepatic transcriptomic analysis revealed distinct differences between QPD and GQD in their gene expression profiles. PCA showed that both interventions significantly reshaped the liver transcriptome, indicating that each formula exerted a marked regulatory effect at the transcriptional level. Compared with GQD, QPD induced broader transcriptomic alterations, as reflected by a greater number of differentially expressed genes relative to the model group. KEGG pathway enrichment analysis further suggested that the two formulas influenced different biological processes. DEGs modulated by GQD were mainly associated with retinol metabolism, bile secretion, chemical carcinogenesis, and arachidonic acid metabolism, which are largely related to metabolic detoxification and inflammatory regulation. In contrast, DEGs regulated by QPD were predominantly enriched in drug metabolism, DNA replication, steroid hormone biosynthesis, and the AGE-RAGE signaling pathway in diabetic complications, indicating a closer association with pathways involved in metabolic regulation and diabetes-related pathological processes. Together, these findings suggest that QPD and GQD may exert anti-diabetic effects through partly distinct transcriptional regulatory patterns, with QPD showing a stronger association with diabetes-related pathway modulation at the hepatic transcriptomic level.

When the gut microbiota, serum metabolomic, and hepatic transcriptomic findings are considered together, and further viewed in light of the correlation analysis, the data suggest that the anti-diabetic response observed in this study is unlikely to result from isolated changes within a single biological layer. Rather, the three omics datasets appear to reflect a coordinated pattern of host-microbe interaction, in which alterations in gut microbial composition are accompanied by changes in circulating metabolites and, in parallel, by adaptive transcriptional responses in the liver. Such a pattern is biologically plausible, as the intestinal microbiota can influence the host metabolic milieu through microbial metabolites and substrate transformation, whereas these systemic metabolic changes may in turn be linked to hepatic gene regulation. From this perspective, the observed cross-omics associations support the existence of a gut microbiota, serum metabolite, and hepatic gene interaction network that may contribute to the improvement of glucose and lipid homeostasis. However, these relationships remain correlative, and further studies will be required to determine whether the identified associations represent direct mechanistic links.

Chemical composition analysis provided the material basis for the divergent biological effects, with comparisons based on UPLC-Q-TOF-MS relative signal intensities (detector counts) (not absolute concentrations). UPLC-Q-TOF-MS identification revealed that while QPD and GQD shared 292 components, each possessed a substantial number of unique constituents. Semi-quantitative classification, based on relative signal abundance, showed that alkaloids and triterpenoids had higher relative signal intensities in GQD than in QPD, whereas QPD exhibited elevated relative signal abundances glycosides, and organic acids. Semi-quantitative profiling further specified that GQD was characterized by high relative signal intensities for alkaloids from Coptis chinensis (e.g., berberine, palmatine) and flavonoid glycosides (e.g., baicalin, wogonoside). In contrast, QPD was enriched with elevated relative signal intensities for isoflavonoids (e.g., daidzin, mirificin, daidzein) and triterpenoids (e.g., glycyrrhizic acid, ganoderic acid S). The elevated relative abundance of glycosides and organic acids in QPD (based on signal intensity) may be closely related to its significant enrichment of SCFA-producing bacteria and modulation of the gut microbiota, as these components often act as prebiotics or microbial metabolic substrates. The high relative signal intensity of alkaloids (e.g., berberine) in GQD, known for their direct antimicrobial and anti-inflammatory activities, may explain its different, sometimes inhibitory, regulatory pattern on the gut microbiota. These distinctive components and their proportional differences likely constitute the chemical basis for the divergent regulatory patterns of QPD and GQD observed at the levels of gut microbiota, serum metabolome, and hepatic transcriptome.

It is important to acknowledge a limitation of the present study regarding safety assessment. While QPD is composed of food-grade homologous ingredients with a history of human consumption, we did not measure specific biochemical markers of hepatic or renal function (such as ALT, AST, creatinine) that would empirically support claims of safety, particularly for long-term use. Although no overt signs of toxicity or adverse effects were observed during the 6-week intervention period, the absence of such data precludes definitive conclusions about the safety of QPD for chronic administration. Future studies incorporating comprehensive toxicological evaluations, including serum biochemistry and histopathological examination of major organs, are necessary to establish the safety profile of QPD and to validate its potential as a long-term dietary intervention for T2DM management.

## 4. Materials and Methods

### 4.1. Chemicals and Reagents

All dried herbal substances used in the experiments were commercially obtained from Beijing Tongrentang Co., Ltd. (Beijing, China), including: *Pueraria lobata*, *Scutellaria baicalensis*, *Coptidis Rhizoma*, *Glycyrrhiza uralensis*, *Astragalus membranaceus*, and *Eriobotrya japonica Thunb.* leaves. Male db/db (C57BLKS/J-Leprdb) mice and db/m (C57BLKS/J-Leprdb/+) mice were purchased from Hangzhou Ziyuan Laboratory Animal Technology Co., Ltd. (Hangzhou, China). The standard chow diet was obtained from Jiangsu Xietong Pharmaceutical Bioengineering Co., Ltd. (Nanjing, China). The mouse insulin ELISA kit was purchased from CUSABIO Life Sciences (College Park, MD, USA).

### 4.2. Decoction Preparation

GQD was prepared in strict accordance with the classical formulation documented in the Shang Han Lun [31]. The initial herbal mixture consisted of *Puerariae Radix* (8 parts), *Coptidis Rhizoma* (3 parts), *Scutellariae Radix* (3 parts), and *Licorice* (2 parts). The mixture was subjected to triple decoction. For each extraction cycle, the herbs were simmered gently in eight times the volume (*v*/*w*) of distilled water at 95 °C for 1 h. After each cycle, the decoction was sequentially filtered through gauze and a 200-mesh sieve. The filtrates from all three extractions were pooled and concentrated under reduced pressure using a rotary evaporator with a water bath temperature of 60 °C. The resulting concentrate was frozen at −80 °C and subsequently lyophilized to obtain a stable freeze-dried powder ready for use.

The FMH-alternative decoction (QPD) was prepared using the identical extraction and processing procedure as GQD for methodological consistency. To maintain the original proportional framework of GQD for formulation standardization, Scutellariae Radix and Coptidis Rhizoma in the classic GQD were replaced with an equal weight of Astragali Radix and *Eriobotrya japonica* Thunb. leaves, respectively. This equal-weight substitution was for formulation proportional consistency only and does not indicate phytochemical or pharmacological equivalence between the original and substitute herbs. Consequently, the QPD formula consisted of *Pueraria lobata* (8 parts), *Astragalus membranaceus* (3 parts), *Eriobotrya japonica* Thunb. leaves (3 parts), and *Glycyrrhiza uralensis* (2 parts).

### 4.3. Animal Experiments

All experimental procedures involving animals were approved by the Animal Care and Use Committee of Jiangnan University (Approval No. JN. No 20210630c0701013[274]) and conducted in accordance with the relevant institutional guidelines. Seven-week-old male db/db mice and their lean littermate db/m mice were acclimatized for one week under specific pathogen-free conditions with a 12 h light/dark cycle and free access to food and water. Forty db/db mice with random blood glucose levels above 16.7 mmol/L were selected as the T2DM model and randomly assigned to five groups (*n* = 8 per group): model control (Mod, saline), metformin (Met, 200 mg/kg BW), GQD (11.8 g/kg BW), QPD-L (7.3 g/kg BW), and QPD-H (11.8 g/kg BW). In addition, 10 db/m mice were used as the normal control group (Con, saline).

The doses of GQD and QPD were determined based on the corresponding clinical raw herb doses used for diabetes treatment. The clinical reference doses for the low- and high-dose formulae were 2.1 and 3.4 g/kg, respectively, calculated on a body weight basis. These doses were converted to mouse-equivalent raw herb doses according to the body surface area normalization method using the equation: mouse dose = human dose × 37/3, where 37 and 3 are the Km factors for adult humans and mice, respectively. The resulting mouse-equivalent raw herb doses were 25.9 g/kg for the low-dose formula and 42.0 g/kg for the high-dose formula. Given an extraction yield of 28.2%, the final administered doses of freeze-dried extract were 7.3 g/kg for QPD-L and 11.8 g/kg for both GQD and QPD-H. All treatments were administered by oral gavage once daily for six consecutive weeks. At the end of the intervention, 24 h urine samples were collected using metabolic cages. After a final 12 h fast, blood and tissues including liver, pancreas, kidney, and colon were collected for subsequent biochemical, metabolomic, and histological analyses.

### 4.4. H&E Staining and Histopathology Analysis

Tissue sections prepared from paraffin-embedded samples were subjected to a standard hematoxylin and eosin (H&E) staining procedure, which included dewaxing, rehydration through a graded ethanol series, and staining. The morphology of the stained sections was visualized and captured using a bright-field microscope (E100, Nikon, Tokyo, Japan). Histopathological assessment was performed semi-quantitatively. The degree of hepatic steatosis and inflammation was evaluated according to the established nonalcoholic fatty liver disease activity score (NAS) system, while pancreatic lesions were scored based on a previously described chronic pancreatitis (CP) classification system [18,32].

### 4.5. Glucose Metabolism Assessment

Fasting blood glucose (FBG) levels were determined at the end of the sixth week of treatment. Following a 12 h fast, blood samples were collected from the tail vein for FBG measurement. In the same week, an oral glucose tolerance test (OGTT) and an analysis of serum fasting insulin (FINS) were also performed. For the OGTT, mice were administered a 20% glucose solution (2 g/kg body weight) by oral gavage, and blood glucose concentrations were measured at 0, 30, 60, 90, and 120 min post-administration. FINS levels in the serum, which was obtained via the retro-orbital bleeding method, were quantified using a commercial mouse insulin enzyme-linked immunosorbent assay (ELISA) kit (Cusabio Biotech Co., Ltd., Wuhan, China). The degree of insulin resistance was then estimated by the homeostasis model assessment of insulin resistance (HOMA-IR) using the following equation:

HOMA-IR = FBG × FIN/22.5(1)

where FBG is fasting blood glucose (mmol/L) and FIN is fasting insulin (μU/mL).

### 4.6. Non-Targeted Metabolomic Profiling

Serum metabolomic profiling was performed using ultra-high-performance liquid chromatography coupled with tandem mass spectrometry (UHPLC-MS/MS). Briefly, 300 µL of each serum sample was mixed with 1200 µL of a pre-chilled extraction solvent (acetonitrile:methanol, 1:4, *v*/*v*) and vortexed for 30 s. The mixture was then incubated at −20 °C for 1 h, followed by centrifugation at 12,000× *g* and 4 °C for 15 min. A 200 µL aliquot of the supernatant was collected and transferred to a clean injection vial. To ensure system stability and data quality, a pooled quality control (QC) sample was prepared by combining equal volumes (10 µL) of all individual serum samples. The QC sample was injected at regular intervals (every 8 experimental samples) throughout the analytical sequence. Metabolite separation was achieved on a Shimadzu LC-30A UHPLC system (Kyoto, Japan) equipped with a Waters ACQUITY UPLC HSS T3 column (Milford, MA, USA, 2.1 mm × 150 mm, 1.8 µm) maintained at 40 °C. The mobile phase consisted of (A) 0.1% formic acid in water and (B) 0.1% formic acid in acetonitrile, with a flow rate of 0.25 mL/min. The gradient elution program was as follows: 0–2 min, 5% B; 2–15 min, 5–95% B; 15–18 min, 95% B; 18–20 min, 95–5% B; 20–25 min, 5% B. Mass spectrometric detection was performed on a TripleTOF 6600+ system (SCIEX, Framingham, MA, USA) operating in both positive and negative electrospray ionization (ESI) modes. The MS parameters were set as follows: ion spray voltage, ±5500 V; source temperature, 500 °C; curtain gas, 35 psi; nebulizer gas (GS1) and heater gas (GS2), 50 psi. Data were acquired in information-dependent acquisition (IDA) mode with a mass range of *m*/*z* 50–1250 for the TOF-MS survey scan and *m*/*z* 25–1250 for the product ion scan.

Raw data files were converted to mzXML format using MSConvert (ProteoWizard, v3.0). Subsequent data preprocessing, including peak picking, retention time alignment, and peak grouping, was performed using the XCMS package (v3.12.0) in R. Features with a relative standard deviation (RSD) > 30% in the QC samples were excluded. The resulting data matrix was normalized by total peak area. To mitigate batch effects, the ComBat function from the sva R package (v3.36.0) was applied. Orthogonal partial least squares-discriminant analysis (OPLS-DA) was conducted using the ropls package (v1.24.0) to visualize group separations. The goodness of fit and predictive ability of the model were evaluated by R2Y and Q2, respectively. In addition, model overfitting was assessed using a 200-permutation test (Figure S1). The *p*-values were further adjusted for multiple testing using the Benjamini–Hochberg False Discovery Rate (FDR) correction. Features with a VIP > 1.0, |log2FC| > 0.263, and an adjusted *p*-value (FDR) < 0.05 were considered statistically significant. Metabolite annotation was conducted by matching the accurate mass (mass error < 10 ppm) and MS/MS fragmentation patterns against a custom spectral library integrating data from public databases (HMDB, METLIN) and in-house standards. According to the Metabolomics Standards Initiative (MSI) guidelines, metabolites identified through such spectral library matching without confirmation by authentic chemical standards are classified as Level 2 (putatively annotated compounds).

### 4.7. Analysis of Gut Microbial Community

Total genomic DNA was extracted from fecal samples collected at the end of the intervention period. The V3-V4 hypervariable regions of the bacterial 16S ribosomal RNA (rRNA) gene were amplified via PCR and subsequently subjected to paired-end sequencing on an Illumina MiSeq platform (Illumina, San Diego, CA, USA). After quality filtering and merging of paired-end reads, the resulting high-quality sequences were denoised and clustered into amplicon sequence variants (ASVs) using the DADA2 pipeline. Taxonomic assignment for the representative ASV sequences was carried out against the Ribosomal Database Project (RDP) 16S rRNA training set, with a minimum confidence threshold of 80% retained for classification. For the samples included in this study (*n* = 18), the sequencing depth ranged from 72,903 to 74,488 clean reads per sample, with an average of 73,716 reads. Alpha diversity was evaluated using the Shannon index to assess microbial diversity within samples. For beta-diversity analysis, the Bray–Curtis dissimilarity matrix was computed based on rarefied ASV abundance data (Figure S2). Principal coordinates analysis (PCoA) was performed to visualize community structure differences. Statistical significance of clustering patterns was evaluated using PERMANOVA (permutational multivariate analysis of variance, 999 permutations) with the adonis2 function from the vegan package, with FDR correction applied to pairwise comparisons between groups. Additionally, analysis of similarities (ANOSIM) was conducted to corroborate PERMANOVA results. Differential abundance analysis was performed at the phylum and genus levels. For taxa with normal distribution (assessed by Shapiro–Wilk test), one-way ANOVA followed by Dunnett’s multiple comparisons test was used; for non-normally distributed taxa, the Kruskal–Wallis test followed by Dunn’s test was applied. Multiple testing correction was performed using the Benjamini–Hochberg FDR method across all tested taxa (adjusted *p* value < 0.05 considered significant). Taxa with prevalence < 10% across samples were excluded from differential abundance analysis to avoid spurious associations driven by rare organisms.

### 4.8. Hepatic Transcriptomic Analysis

Total RNA was isolated from liver tissue using Trizol reagent (Labgic Co., Ltd., Beijing, China)., with quality and integrity verified prior to library construction. Sequencing libraries were prepared and subjected to paired-end sequencing (2 × 150 bp) on an Illumina NovaSeq platform. Raw reads were processed by removing adapter-containing reads, reads with more than 10% unidentified nucleotides (N), and reads in which more than 50% of bases had a quality score ≤ 10, to obtain clean reads. The clean reads were aligned to the mouse reference genome (*Mus_musculus.*GRCm38_release95.genome.fa) using HISAT2, with mapping rates ranging from 95.87% to 97.00%. The mapped reads were assembled using StringTie for transcript reconstruction and expression quantification, and gene-level count values were generated for downstream analysis. Differential expression analysis was performed using the DESeq2 package based on gene count data. Genes with |log2 fold change| ≥ 1 and false discovery rate (FDR) ≤ 0.05 were defined as differentially expressed genes (DEGs). Functional enrichment analysis of DEGs was subsequently performed using the Kyoto Encyclopedia of Genes and Genomes (KEGG) pathway database.

### 4.9. RT-qPCR

Total RNA was isolated from liver tissues using a total RNA isolation reagent (Biosharp, Hefei, China). Reverse transcription and quantitative real-time PCR (RT-qPCR) were performed using a commercial kit (Yeasen, Shanghai, China) on a fluorescence quantitative detection system (BIOER FQD-96A, Hangzhou, China). Three independent biological replicates were analyzed, and relative expression levels of target genes were calculated using the 2^−ΔΔCT^ method. The primer sequences are available upon request.

### 4.10. Identification of Chemical Constituents in GQD and QPD

The chemical constituents of GQD and QPD were identified using ultra-performance liquid chromatography coupled with quadrupole time-of-flight mass spectrometry (UPLC-QTOF-MS). Chromatographic separation was carried out on an ACQUITY Premier I-Class UPLC system (Waters, Milford, MA, USA) equipped with an HSS T3 column (150 mm × 2.1 mm, 1.8 μm) maintained at 40 °C. The mobile phase consisted of (A) 0.1% (*v*/*v*) formic acid in water and (B) 0.1% (*v*/*v*) formic acid in acetonitrile, with a flow rate of 0.25 mL/min and a 2 μL injection volume. Mass spectrometry analysis was performed using a Xevo G3 QTof instrument (Waters, Milford, MA, USA), operating in both positive and negative electrospray ionization (ESI) modes. Raw data were processed and compound identification was conducted using the Waters Connect informatics platform. Chemical components were identified by comparing accurate mass measurements and MS/MS fragmentation pat-terns to comprehensive spectral libraries, including the Natural Products Atlas, the University of Mississippi Botanical Library, and the Waters Traditional Medicine Library, within the UNIFI 1.9.4 Scientific Information System.

### 4.11. Statistical Analysis

All data are expressed as mean ± standard error of the mean (SEM). Statistical analyses were performed using GraphPad Prism version 8.0.2. Differences between two groups were assessed using Student’s *t*-test. For normally distributed data, one-way analysis of variance (ANOVA) followed by an appropriate post hoc test was used, whereas non-normally distributed data were analyzed using the Kruskal–Wallis test followed by an appropriate post hoc test. The specific statistical methods used for each dataset are described in the corresponding Methods subsections and figure legends. For oral glucose tolerance test (OGTT) time-course data obtained from repeated measurements in the same animals, two-way repeated-measures ANOVA with Greenhouse–Geisser correction was applied to evaluate the effects of group, time, and group × time interaction, followed by Bonferroni post hoc tests for pairwise comparisons at individual time points and for the area under the curve (AUC). A value of *p* < 0.05 was considered statistically significant.

## 5. Conclusions

This study systematically compared the antidiabetic effects and molecular mechanisms of the food–medicine homologous formula QPD and the classic formula GQD in type 2 diabetic mice from five integrated dimensions: glucose metabolism and histopathology, gut microbiota, serum metabolome, hepatic transcriptome, and chemical composition. The results demonstrated that both QPD and GQD significantly ameliorated glucose metabolic disorders and alleviated hepatic and pancreatic pathological damage in diabetic mice, with comparable overall efficacy. Regarding mechanistic regulation, both formulas remodeled gut microbiota structure, modulated serum metabolite profiles, and influenced hepatic gene expression; however, marked differences were observed in their regulatory patterns of key bacterial taxa, differential metabolites, and signaling pathways. QPD preferentially modulated metabolic and signaling pathways closely associated with diabetes pathogenesis, whereas GQD exhibited a distinct distribution of regulatory targets. Chemical profiling revealed substantial differences in compound composition, class distribution, and relative abundances between the two formulas, providing a crucial material basis for their divergent pharmacological characteristics.

These findings demonstrate that QPD, formulated based on the food-medicine homologous principle, exerts beneficial effects comparable to those of GQD against T2DM in a mouse model. The edible nature of its components supports its potential suitability for dietary approaches to T2DM management, although its long-term safety profile remains to be established through dedicated toxicological studies. This study not only elucidates the mechanisms underlying QPD’s anti-diabetic activity and supports its translational potential, but also offers a valuable reference framework for the development and optimization of food–medicine homologous formulations. Future studies should further validate these findings in additional experimental models, directly quantify key microbial metabolites, and incorporate functional approaches to clarify the causal relationships among gut microbiota remodeling, metabolic alterations, and host transcriptional responses.

## Figures and Tables

**Table 1:** Semi-quantitative comparison of representative differential components between QPD and GQD based on UPLC-Q-TOF-MS relative signal intensities.

GQD			QPD		
Compound Name	Category	Detector Counts	Compound Name	Category	Detector Counts
Moupinamide	Alkaloid	2,566,699	Daidzin	Isoflavonoid glycoside	1,873,457
Palmatine	Alkaloid	2,251,543	Mirificin	Isoflavonoid glycoside	1,388,829
Chrysophanol glucoside	Anthraquinone glycoside	2,056,623	Aloe emodin 8-glucoside	Anthraquinone glycoside	1,235,448
Wogonoside	Flavonoid glycoside	1,929,186	Glycitin	Isoflavonoid glycoside	1,197,427
Baicalein-6-glucuronide	Flavonoid glycoside	1,724,825	Glycyrrhizic acid	Triterpenoid saponin	939,482
d-Thaliporphine (Thaliporphine)	Alkaloid	1,685,465	Daidzein	Isoflavone aglycone	673,756
Aloe emodin 8-glucoside	Anthraquinone glycoside	1,597,406	Daidzein-4′,7-diglucoside	Isoflavonoid glycoside	614,951
Berberine	Alkaloid	1,507,373	Yuankanin	Flavonoid glycoside	578,636
Mirificin	Isoflavonoid glycoside	1,441,952	Ononin	Isoflavonoid glycoside	449,811
Glycitin	Isoflavonoid glycoside	1,412,460	Ganoderic acid S	Triterpenoid acid	446,218
Decarine	Alkaloid	1,405,241	Kushenol O	Flavonoid	429,848
Glycyrrhizic acid	Triterpenoid saponin	1,040,710	Chrysophanol glucoside	Anthraquinone glycoside	408,779

## Data Availability

The original contributions presented in this study are included in the article/Supplementary Materials. Further inquiries can be directed to the corresponding author.

## Supplementary Materials

The following supporting information can be downloaded at: https://www.mdpi.com/article/10.3390/ph19040530/s1, Figure S1: Permutation test of the OPLS-DA model based on 200 permutations. Figure S2: Alpha and beta diversity analyses of gut microbiota among different groups. (A) Alpha diversity assessed by the Shannon index. (B) Beta diversity, with group differences tested by PERMANOVA.

## Abbreviations

The following abbreviations are used in this manuscript:

T2DM	Type 2 diabetes mellitus
GQD	Gegen Qinlian Decoction
FMH	food and medicine homologous
QPD	Qige Pipa Decoction
NAS	liver disease activity score
CP	chronic pancreatitis
FBG	Fasting blood glucose
OGTT	oral glucose tolerance test
FINS	fasting insulin
Mod	model
Con	control
HOMA-IR	homeostatic model assessment for insulin resistance
AUC	area under the curve
SCFA	short-chain fatty acid
