# Supplementary material for: Beneficial Effects of Gegen Qinlian Decoction and Its Food–Medicine Homologous Alternative Formulas Against Type 2 Diabetes Mellitus: Insights from Multi-Omics Analysis
Source: Pharmaceuticals (Basel). 2026 Mar 25;19(4):530. doi: 10.3390/ph19040530 (PMC13119408; doi:10.3390/ph19040530)

Beneficial Effects of Gegen Qinlian Decoction and Its Food–Medicine Homologous Alternative Formulas Against Type 2 Diabetes Mellitus: Insights from Multi-omics Analysis

Yao Chen[1,2], Dandan Ma[1,2], Qiuming Chen[1,2], Maomao Zeng[1,2], Jie Chen[1,2] and Zhiyong He[1,2,*]

1. State Key Laboratory of Food Science and Resources, Jiangnan University, Wuxi 214122, China

2. School of Food Science and Technology, Jiangnan University, Wuxi 214122, China

*Corresponding author: zyhe@jiangnan.edu.cn (Z. He), chenjie@jiangnan.edu.cn (J. Chen)

Supplementary Figures:

Figure S1. Permutation test of the OPLS-DA model based on 200 permutations.

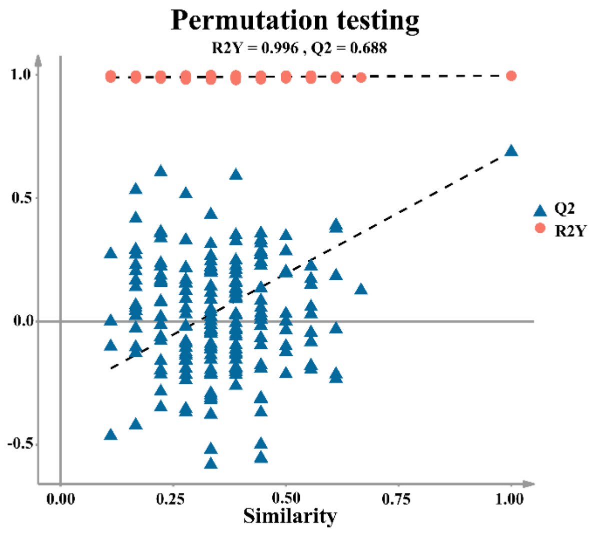

Figure S2. Alpha and beta diversity analyses of gut microbiota among different groups. (A) Alpha diversity assessed by the Shannon index. (B) Beta diversity, with group differences tested by PERMANOVA.

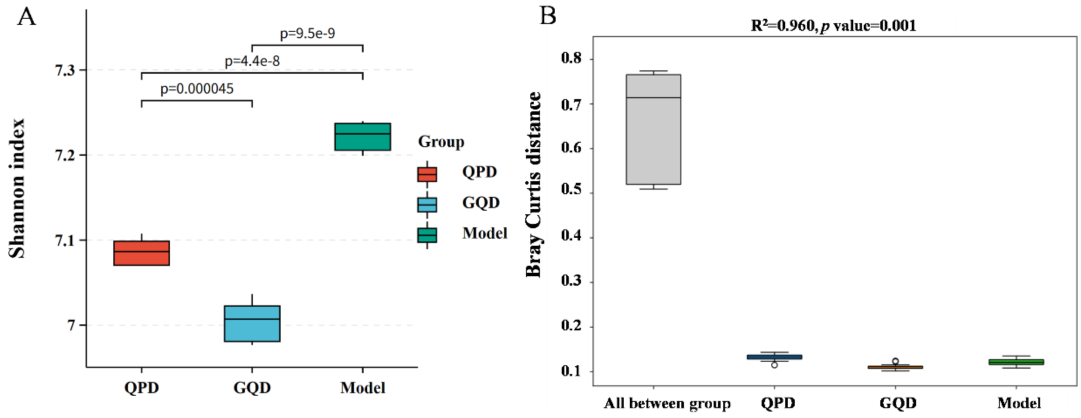

Supplement: Supplementary file 1 [file pharmaceuticals-19-00530-s001.zip › pharmaceuticals-4200971-supplementary.pdf]
